# Differential Regulation of Gene and Protein Expression by Zinc Oxide Nanoparticles in Hen’s Ovarian Granulosa Cells: Specific Roles of Nanoparticles

**DOI:** 10.1371/journal.pone.0140499

**Published:** 2015-10-13

**Authors:** Yong Zhao, Lan Li, Peng-Fei Zhang, Wei Shen, Jing Liu, Fen-Fang Yang, Hong-Bo Liu, Zhi-Hui Hao

**Affiliations:** 1 College of Chemistry and Pharmaceutical Sciences, Qingdao Agricultural University, Qingdao, 266109, P. R. China; 2 Key Laboratory of Animal Reproduction and Germplasm Enhancement in Universities of Shandong, Qingdao, 266109, P. R. China; 3 Core Laboratories of Qingdao Agricultural University, Qingdao, 266109, P. R. China; Institute for Materials Science, GERMANY

## Abstract

Annually, tons and tons of zinc oxide nanoparticles (ZnO NPs) are produced in the world. And they are applied in almost all aspects of our life. Their release from the products into environment may pose issue for human health. Although many studies have reported the adverse effects of ZnO NPs on organisms, little is known about the effects on female reproductive systems or the related mechanisms. Quantitative proteomics have not been applied although quantitative transcriptomics have been used in zinc oxide nanoparticles (ZnO NPs) research. Genes are very important players however proteins are the real actors in the biological systems. By using hen’s ovarian granulosa cells, it was found that ZnO-NP-5μg/ml and ZnSO_4_-10μg/ml treatments produced the same amount of intracellular Zn and resulted in similar cell growth inhibition. And NPs were found in the treated cells. However, ZnO-NP-5μg/ml specifically regulated the expression of genes and proteins compared with that in ZnSO_4_-10μg/ml treatment. For the first time, this investigation reports that intact NPs produce different impacts on the expression of genes and proteins involved in specific pathways compared to that by Zn^2+^. The findings enrich our knowledge for the molecular insights of zinc oxide nanoparticles effects on the female reproductive systems. This also may raise the health concern that ZnO NPs may adversely affect the female reproductive systems through regulation of specific signaling pathways.

## Introduction

Nanoparticles (NPs) have at least one dimension less than 100 nm with unique characteristics compared to their corresponding bulk materials [1]. These characteristics include small size, large surface area to volume ratio, typical smoothly scaling properties, and others. These specific characteristics make Zinc oxide (ZnO) NPs useful nano-materials as they have been applied in numerous industrial products (e.g., plastics, ceramics, glass, cement, rubber, paints and pigments). Most impressively, ZnO NPs are widely used metal oxide NPs in medical disinfection. They were found to inhibit the growth of bacterial [2], fungal [3] and virus [4, 5]. And also they have been used in personal care products like sunscreens and cosmetics due to their excellent UV absorption and reflective properties [6]. Furthermore, the small size also helps ZnO NPs readily be absorbed into biological systems through cellular uptake and the interaction with internal or membrane molecules. It has been found that feeding chicken with ZnO NPs could improve growth performance and anti-oxidative capabilities in broilers [7]. However, numerous studies have reported that ZnO NPs caused adverse effects on organisms such as toxicity on *Daphnia magna* [8, 9], zebrafish embryos [10], rat reproductive development [11], mouse spermatogenesis [12], human hepatocyte cells, immune cells and others [13–15]. Even though some studies have investigated the effect of ZnO NPs on reproductive system [11, 12], it is unknown about the molecular insights of ZnO NPs on female reproductive systems. Actually, changes in protein expression after ZnO NPs exposure or specific pathways regulated by NPs have not been reported. Brun found that the effect of ZnO NP was solely related to Zn^2+^ [10]. However, Chen and Poynton reported that the effect of ZnO NPs exposure was related to both NPs and Zn^2+^ [9, 16].

Domestic chickens (*Gallus gallus*) are important model organisms because they bridge the evolutionary gap between mammals and other vertebrates [17]. And their genome was sequenced firstly among all the agricultural animals. Due to the experimental advantages of *in ovo* embryogenesis, the embryo of the chicken is a useful vertebrate system for the developmental biologists. Furthermore, chickens are inexpensive and easy to handle which makes them an excellent animal model for researches. Chicken oocytes develop in three major phases: (1) developing to white follicles without yellow yolk inside (increasing size from 60μm to 2-3mm in diameter) taking a few months; (2) developing to small yellow follicles with yolk inside (6-7mm in diameter); (3) developing to large follicles (5–8 large follicles with size from 8-30mm in diameter) with the largest one (30mm in diameter) ovulating each day. The large follicles contain about 30–50 million granulosa cells (GCs) depending on the size [18]. Therefore the large follicles from one hen are enough for a number of biochemical analyses [19]. And also the granulosa cells of chicken are steroidogenic hormone production cells and they play very important roles in oocyte development and early embryogenesis because they are the closest cells to germ cells with transporting nutrition and producing other factors for oocyte growth. These are similar for chickens and mammals [20–22]. Therefore, the chicken ovarian granulosa cells (GCs) were chosen as a model in this study to investigate the effects and the molecular insights of ZnO NPs on female reproductive systems. The hens used in this investigation were from Jinghong-1 strain which were developed by Beijing Huadu Yukou Poultry Industry Co. Ltd. These hens have lots of advantages such as laying eggs at early age, high production and low consumption. ZnO NPs have been reported to result in adverse effect on organisms and to change the expression of genes related to cytoskeletal transport, cellular respiration, and reproduction in *Daphnia magna* [9]. Do ZnO NPs alter protein expression? It is unknown yet. The hypothesis of current study was that intact NPs instead of Zn^2+^ might regulate the specific signaling pathways in female reproductive systems. ZnSO_4_ was used as a control (sole Zn^2+^ provider). Quantitative transcriptomics and quantitative proteomics were used in this investigation with two major analysis methods Gene Ontology (GO) analysis and KEGG (Kyoto Encyclopedia of Genes and Genomes) analysis. GO Consortium is a dynamic, controlled vocabulary which is applied to all eukaryotes for explaining the roles of gene and protein in cells. There are three independent ontologies (http://www.geneontology.org): biological process, molecular function and cellular component. KEGG (http://www.kegg.jp/), a database resource, aims to help understanding high-level functions and utilities of the biological systems (cells, organisms) from molecular-level information (especially large-scale molecular datasets generated by genome sequencing and protein sequencing). It was found that ZnO-NP-5μg/ml specifically regulated the expression of genes and proteins in GCs. Due to wide usage, releasing from products to environment and easily getting into human body, ZnO NPs may accumulate in our organs and pose adverse effect on the female reproductive systems.

## Materials and Methods

### ZnO nanoparticles Characterization

ZnO NPs were synthesized by Beijing DK nano technology Co. LTD (Beijing, P. R. China). The morphology, size, and agglomeration were characterized by transmission electron microscopy (TEM; JEM-2100F, JEOL Inc., Japan) and dynamic light scattering (DLS) particle size analyzer (Nano-Zetasizer-HT, Malvern Instruments, Malvern, UK).

### Granulosa Cells (GC) Isolation and Culture

This investigation was carried out in strict accordance with the recommendations in the Guide for the Care and Use of Laboratory Animals of the National Institutes of Health. The protocol was approved by the Committee on the Ethics of Animal Experiments of Qingdao Agricultural University. 30-40weeks old Jinghong-1 laying hens were obtained from Maochangyuan Co. (Qiangdao, China). The hens were terminated, and granulosa cells were isolated from large pre-ovulatory follicles (POFs) as described previously with minor modifications [20]. Briefly, the sheets containing perivitelline layer and granulosa cell from follicles were carefully separated from yolk. Then the sheets were washed in cold, sterile PBS twice and cut to small pieces. The granulosa cells (GCs) were separated by mechanically pipetting up and down for 2 min. The GCs suspension was filtered and the cell pellets were washed twice by cold PBS and once in blank M199 medium (Cat. No: 31100–019; Gibco^®^, Life Technologies, Carlsbad, CA, U.S.A.) followed by 4-min centrifugation at 250 x*g* at room temperature. Cell viability of GC was determined to be >95% after their isolation. The cells were cultured under standard conditions (37°C, 5% CO_2_) in M199 medium supplemented with penicillin (50 U/ml), streptomycin (50 mg/ml), 10% fetal bovine serum (FBS; Cat. No: 10099–41; Gibco^®^ Life Technologies), and 1% of Insulin-Transferrin-Selenium-A Supplement (ITS) (Catalog no. 51300–044; Gibco^®^, Life Technologies.).

### Detection of ZnO NPs in Cells by Transmission Electron Microscopy (TEM) and Energy Disperse Spectroscopy (EDS)

The sample preparation for TEM and EDS analysis were followed the publication by Yamashita [23]. Briefly, ZnO-NP-5μg/ml treated GCs pellets were collected and fixed for 2 h in 2% glutaraldehyde made in sodium phosphate buffer (pH 7.2). The specimens were washed extensively to remove the excess fixative and subsequently post-fixed in 1%OsO_4_ for 1h in the dark. After extensive washes in phosphate buffer, the cells were dehydrated in an increasing graded series of ethanol and infiltrated with increased concentration Spur’s embedding medium in propylene epoxide. Then the specimens were polymerized in embedding medium 12h at 37°C, 12h at 45°C and 48h at 60°C. Fifty nanometer were cut on a Leica Ultracut E equipped with a diamond knife (Diatome, Hatfield, PA), and collected on form var-coated, carbon-stabilized molybdenum (Mo) grids. The section-containing grids were stained with uranyl acetate, allowed to air dry overnight, and imaged on a JEM-2010F TEM (JEOL Ltd., Japan). ZnO nanoparticles in the cells were confirmed by X-Max^N^ 80 TLE EDS (Oxford Instruments, U.K.).

### Determination of ZnO NPs Effects on Cell Viability

GCs were plated in 96-well plates at a density of 5 x 10^4^ cells /well. Then GCs were treated with different concentrations (based on Zn; 1, 5, 7.5, 10, 12.5 15, 20, 30μg/ml) of ZnO NPs or ZnSO_4_ (Cat. No: Z1001, Sigma-Aldrich Co. LLC in China, Beijing, P.R. China) for 24h. Before treatments the cells were recovered and grown for 48h. After 24h treatments, the cells were washed with fresh basic medium (No FBS or antibiotics) and then the cell viability was determined by the reported method using a colorimetric assay with MTT [3-(4, 5)-dimethylthiazol-2, 5-diphenyltetrazolium bromide; Cat. No: M5655, Sigma-Aldrich] [24].

### Measurement of Zn Concentration in Cultured Cells

The GCs were plated in 6-well plates and grown for 2 days then the cells were treated with different concentration (based on Zn; 1, 5 and 10μg/ml) of ZnO NP or ZnSO_4_ for 24h. About 2×10^6^ cells/treatment were lysed in 500μL of 0.4% Triton X-100 in PBS, then the lysate was diluted to 2mL with 0.1% Triton X-100. Samples were determined by inductively coupled plasma optical emission spectroscopy (ICP-OES, Optima 2100, Perkin-Elmer, Shelton, CT, USA) [25].

### Cell-cycle analysis

The cell-cycle distribution for GCs was determined by flow cytometry [26]. After 24h treatment with NPs, GCs were trypsinized and counted. Then 1 × 10^6^ cells were fixed in ethanol at 4°C overnight, then the cells were resuspended in PBS (pH 7.4), and incubated with propidium iodide (50μg/mL; Sigma; for 1h) at room temperature in the dark. Fluorescence was measured at 585 nm with a FacsCalibur cytometer (BD Biosciences, San Diego, USA). A single-cell gate was used to exclude aggregated cells, and 10,000 gated events were collected for each sample. The cell-cycle profile was analyzed with Cell Quest Pro software (BD Biosciences).

### Effects of ZnO NPs on mRNA Expression Determined by Quantitative Transcriptomics (RNA-seq transcript profiling) and mRNA q-RT-PCR

#### RNA-seq transcript profiling

Transcriptomics were analyzed as described [27, 28]. Briefly, total RNA was isolated by TRIzol Reagent (Invitrogen, U.S.A.) and purified with PureLink® RNA Mini Kit (Cat: 12183018A; Life Technologies) following the manufacturer’s protocol. The total RNA samples were first treated with DNase I to degrade any possible DNA contamination. Then the mRNA was enriched by using the oligo(dT) magnetic beads. Mixed with the fragmentation buffer, the mRNA was fragmented into short fragments (about 200 bp). Then the first strand of cDNA was synthesized by using random hexamer-primer. Buffer, dNTPs, RNase H and DNA polymerase I were added to synthesize the second strand. The double strand cDNA was purified with magnetic beads. And 3'-end single nucleotide A (adenine) addition was then performed. Finally, sequencing adaptors were ligated to the fragments. The fragments were enriched by PCR amplification. During the QC step, Agilent 2100 Bioanaylzer and ABI StepOnePlus Real-Time PCR System were used to qualify and quantify the sample library. The library products were ready for sequencing via Illumina HiSeqTM 2000. The reads were mapped to reference gene using SOAPaligner (Version 2.20) with a maximum of two nucleotide mismatch allowed at the parameters of “-m 0 -x 1000 -s 40 -l 35 -v 3 -r 2”. The read number of each gene was transformed into RPKM (Reads Per Kilo bases per Million reads), and then differently expressed genes were identified by the DEGseq package using the MARS (MA-plot-based method with Random Sampling model) method. The threshold was set as FDR≤ 0.001 and an absolute value of log_2_Ratio≥ 1 to judge the significance of gene expression difference.

#### mRNA q-RT-PCR

Total RNA was isolated as described as above. RNA concentration was determined by Nanodrop 3300 (ThermoScientific, Wilmington, DE). Two micrograms of total RNA was used to make the first strand complementary DNA (cDNA; in 20μl) using RT2 First Strand Kit (Cat. No: AT311-03, Transgen Biotech, P. R. China) following the manufacturer’s instructions. The generated first-strand cDNAs (20μl) was diluted to 150μl with double-deionized water (ddH2O). Then, 1μl was used for one PCR reaction (in a 96-well plate). Each PCR reaction (12μl) contained 6μl of qPCR Master Mix (Roche, German), 1μl of diluted first-stand cDNA, 0.6μL primers (10μM), and 4.4μl of ddH2O. The primers for qPCR analysis were synthesized by Invitrogen and present in S1 Table. The qPCR was conducted by the Roche LightCycler^®^ 480 (Roche, German) with the following program-step 1: 95°C, 10 min; step 2: 40 cycles of 95°C, 15 s; 60°C, 1 min; step 3: dissociation curve, step 4: cool down. Three or more independent experiment samples were analyzed.

### Effects of ZnO on Proteins Detected by Quantitative Proteomics (Isobaric Tag for Relative and Absolute Quantitation, iTRAQ) and Western Blotting

#### iTRAQ

Proteomics were analyzed according to the previous reports [29, 30]. GCs were lysed in lysate buffer containing PMSF (1mM), EDTA (2mM), DTT (10mM). Proteins, 100μg each sample, were digested with Trypsin Gold (protein: trypsin = 20:1) at 37°C for 4 h. The iTRAQ labeling of peptide samples was performed using iTRAQ Reagent 8-plex Kit according to the manufacturer’s protocol. The iTRAQ labeled peptides were fractionated by SCX chromatography using Shimadzu LC-20AB HPLC Pump system. Data acquisition was performed with a TripleTOF 5600 System (AB SCIEX, Concord, ON) fitted with a Nanospray III source (AB SCIEX, Concord, ON) and a pulled quartz tip as the emitter (New Objectives, Woburn, MA). The MS was operated with a RP of greater than or equal to 30 000 FWHM for TOF MS scans. For IDA, survey scans were acquired in 250 ms and as many as 30 product ion scans were collected if exceeding a threshold of 120 counts per second (counts/s) and with a 2+ to 5+ charge-state. For protein identification, the filters were set as significance threshold *P*, 0.05 (with 95% confidence) and ion score or expected cutoff less than 0.05 (with 95% confidence). For protein quantitation, “median” was chosen for the protein ratio type; the minimum precursor charge was set to 2+ and minimum peptides were set to 2; only unique peptides were used to quantify proteins. The median intensities were set as normalization, and outliers were removed automatically.

#### Western blotting

GCs were lysed in RIPA buffer containing the protease inhibitor cocktail from Sangong Biotech, Ltd. (Shanghai, P.R. China). Protein concentration was determined by BCA kit (Beyotime Institute of Biotechnology, Shanghai, P. R. China). Rabbit anti-glyceraldehyde 3-phosphate dehydrogenase (GAPDH) (Cat no.: BA2913, Boster Co., P. R. China) was used as a loading control. The following primary antibodies (Abs) were purchased from Abcam Trading Company Ltd. (Shanghai, P.R. China): rabbit polyclonal anti-CRIP1Ab (Cat no.: ab-102123), rabbit polyclonal anti-DHRS7B Ab (Cat no.: ab-98880), goat polyclonal anti-NCAM2 Ab (Cat no.: ab-134785), rabbit monoclonal anti-PIWIL1 Ab (Cat no.: ab94917), rabbit polyclonal anti-DDX4 Ab (Cat no.: ab13840) and rabbit polyclonal anti-VGT2 (Cat no.: ab193340). Rabbit polyclonal anti-CSRP2 Ab (Cat no.: bs-12946R) and NDRG1 Ab (Cat no.: bs-1584R) was purchased from Shanghai Enzyme-linked Biotechnology Co., Ltd. Secondary donkey anti-goat Ab (Cat no.: A0181) was purchased from Beyotime Institute of Biotechnology (Shanghai, P.R. China), and goat anti-mouse (Cat no.: A24512) and goat anti-rabbit (Cat no.: A24531) Abs were bought from Novex^®^ by life technologies (USA). Thirty micrograms of total protein per sample was loaded onto 10% SDS–polyacrylamide gel electrophoresis gels for Western blot analysis. The gels were transferred to Polyvinylidene Fluoride (PVDF) membrane in cool room (4°C) at 300mA for 3h. Then, the membranes were blocked with 5% BSA for 1 h at room temperature, followed by three washes with 0.1% Tween 20 in TBS (TBST). The membranes were incubated with primary Abs diluted with 1:500 in TBST with 1% BSA overnight at 4°C. After three washes with TBST, the blots were incubated with the HRP-labeled secondary goat anti-mouse, goat anti-rabbit, or donkey anti-goat Ab respectively for 1 h at room temperature. Then, the blots were imaged after three washes.

### Statistical analyses

The q-RT-PCR was statistically analyzed using proprietary software from SABiosciences online support. Other results are expressed as mean ± SEM. Differences are considered significant at *p* < 0.05 using Student’s *t* test or ANOVA.

## Results

### Characteristics of ZnO NPs

**Fig 1:**
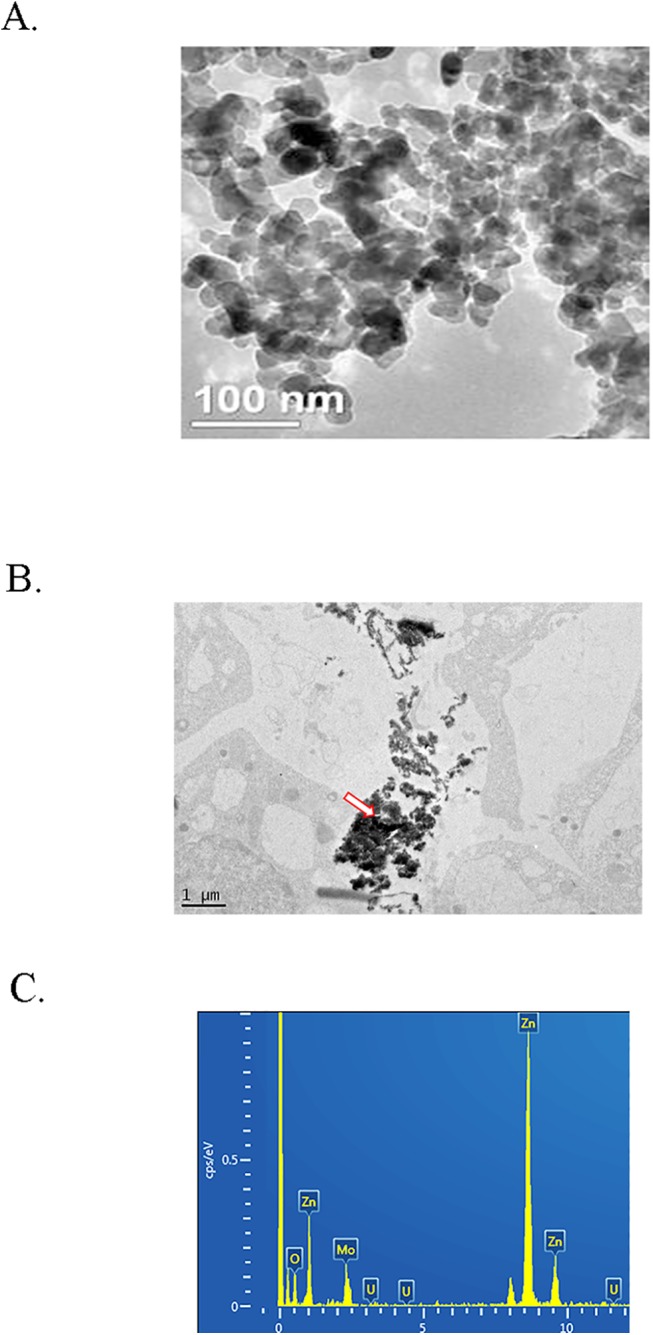
Characteristics of ZnO NPs, and NPs in GCs. A. TEM photo of ZnO NPs. B. TEM image of ZnO NPs in GCs indicated by the red-white arrow. C. EDS picture of ZnO NPs in GCs, three standard Zn peaks shown.

Fig 1A presents the ultra-structures of NPs. The particles are nearly spherical and the color is milk white. The size and the surface area are about 30nm and 50m^2^/g, respectively. And the density of the NPs is 5.606g/cm^3^.

### ZnO NPs found in GCs

After 24h ZnO-NP-5μg/ml treatment, TEM was used to detect ZnO NPs in the treated cells. NPs were found in ZnO-NP-5μg/ml treated GCs (Fig 1B; indicated by red-white arrow). The NPs were confirmed by Energy Disperse Spectroscopy (EDS) with Zn (Fig 1C). Three standard Zn peaks were present.

### Effect of ZnO NPs on the Growth of Granulosa Cells (GCs)

**Fig 2:**
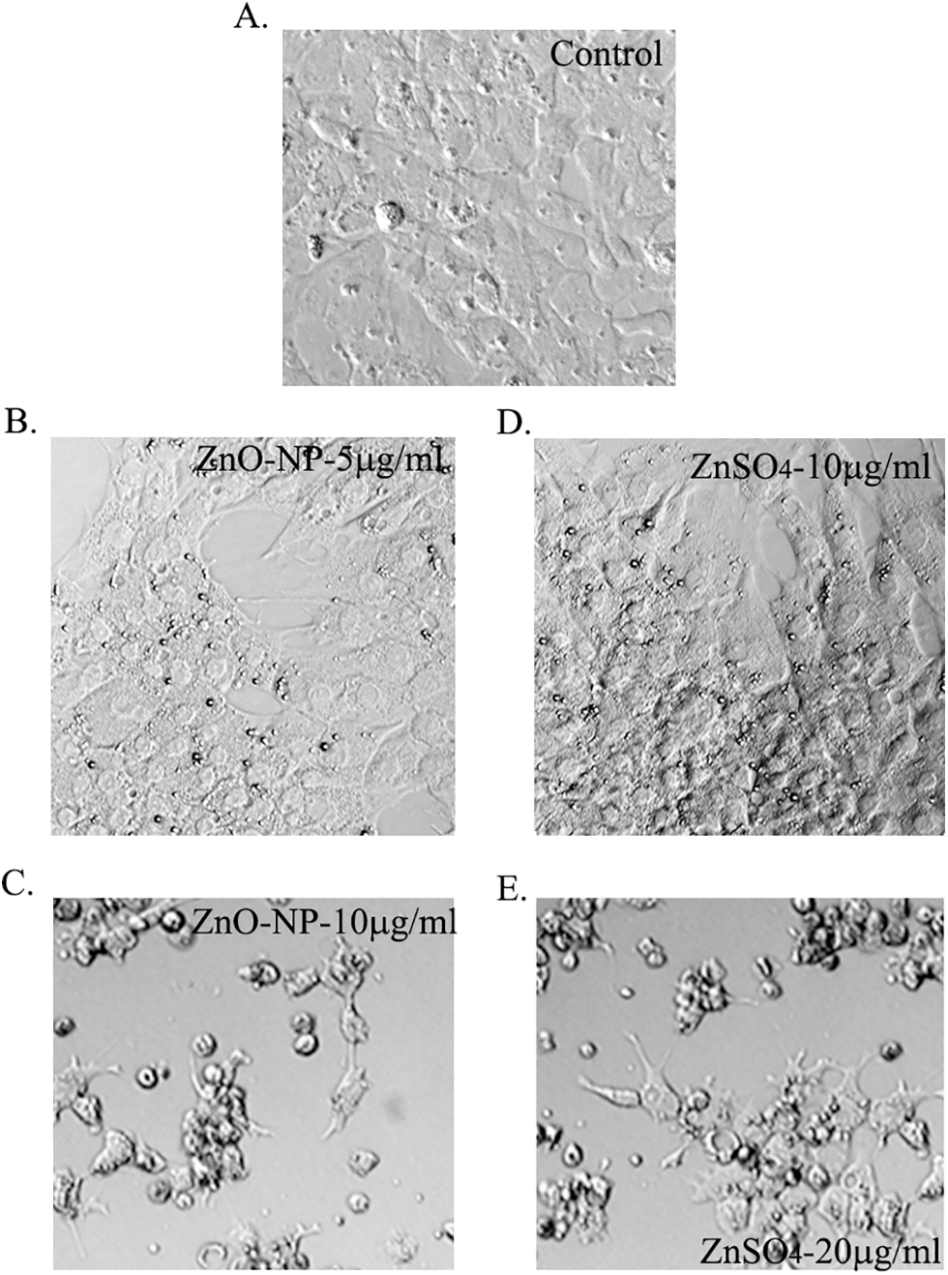
Effects of ZnO NPs and ZnSO_4_ on GCs growth. A. Photo for control GCs (with just growth medium); B. Photo of ZnO-NP-5μg/ml treated GCs after 24h treatment; C. Photo of ZnO-NP-10μg/ml treated GCs after 24h treatment; D. Photo of ZnSO_4_-10μg/ml treated GCs after 24h treatment; E. Photo of ZnSO_4_-20μg/ml treated GCs after 24h treatment.

Since NPs were found in treated GCs, the growth, the basic effect of ZnO NPs on granulosa cells, was investigated. Both ZnO NPs and ZnSO_4_ inhibited the growth of GCs after 24h treatment (Fig 2). However, ZnO NPs caused higher cell growth inhibition than ZnSO_4_. The half maximal inhibitory concentration (IC_50_) for ZnO NPs and ZnSO_4_ were 7.3μg/ml and 11.1μg/ml (based on Zn) respectively. Both ZnO-NP-5μg/ml (Fig 2B) and ZnSO_4_-10μg/ml (Fig 2D) treatments produced similar cell growth inhibition (about 27%).

### Concentration of Zn in GCs

Because same concentration of ZnO NPs and ZnSO_4_ produced different cell growth inhibition, the concentration of Zn in the treated cells was investigated. The concentrations of Zn in GCs after 24h treatment were 1.08±0.19 and 1.26±0.17μg/mg protein for ZnO-NP-5μg/ml and ZnSO_4_-10μg/ml treatments respectively (No significant difference). Since these two treatments produced same intracellular Zn concentration and resulted in similar cell growth inhibition, they were employed for further investigation.

### Effects of ZnO NPs on the Expression of Genes

**Fig 3:**
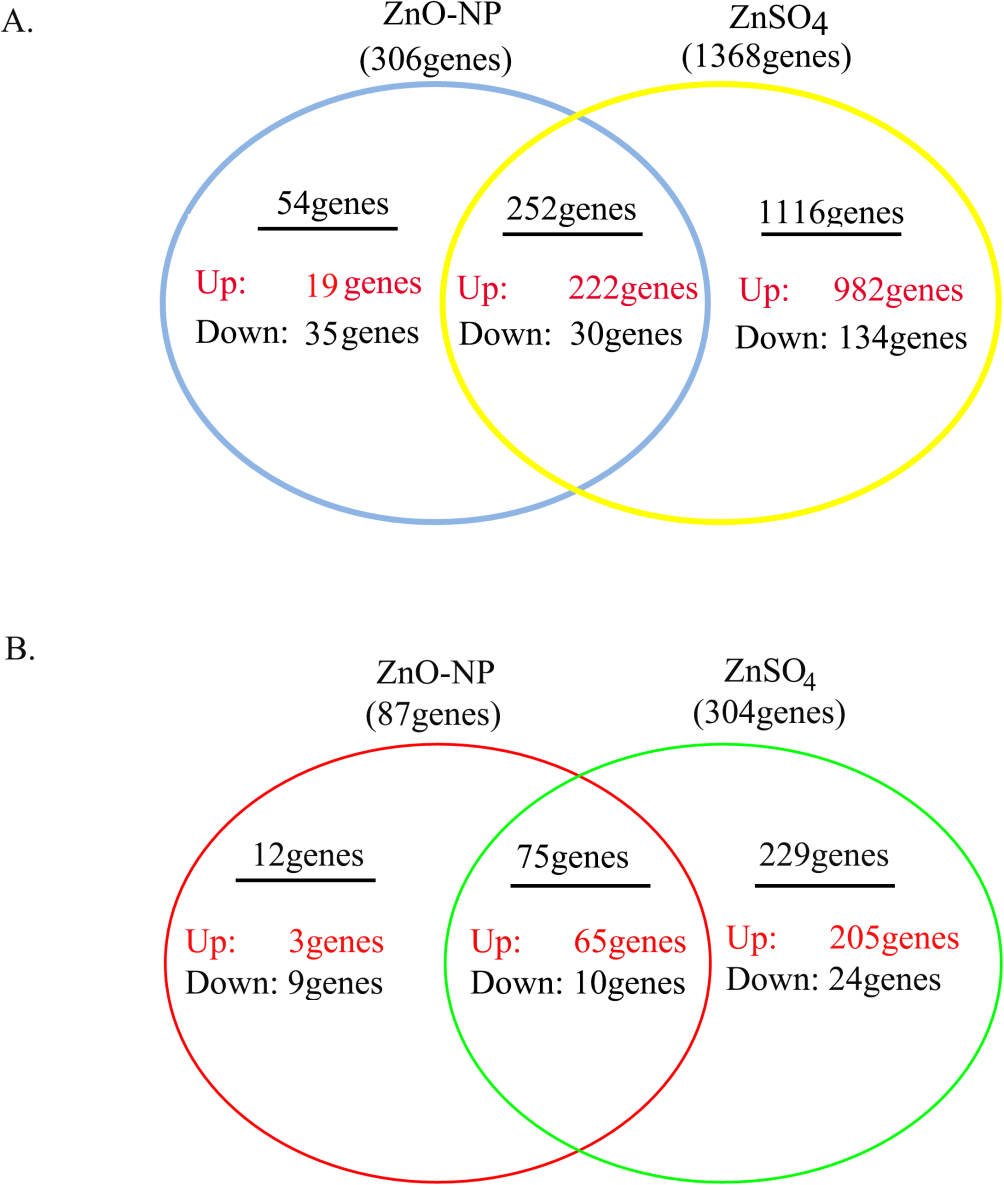
Effects of ZnO-NP-5μg/mL and ZnSO4-10μg/mL treatments on GCs mRNAs expression. A. Summary of number of mRNAs regulated by ZnO-NP-5μg/mL and/or ZnSO4-10μg/mL, and the same sets of mRNAs regulated by the two treatments, and the different sets of mRNAs regulated by each treatment. B. The regulated mRNAs related to development, growth and reproduction by these two treatment groups. The red color meant the mRNAs were up regulated, black means the mRNAs were down regulated. The number was the log_2_ value, cut off at log_2_Ratio≥1 or log_2_Ratio≤-1. They were significant regulated.

In the quantitative transcriptomics (RNA-seq transcript profiling) analysis, 13537 genes in ZnO-NP-5μg/ml and 13829 genes in ZnSO_4_-10μg/ml treated GCs were identified and quantified (Table 1). Totally, the expression of 306 genes were changed by ZnO-NP-5μg/ml, and 1368 genes were altered by ZnSO_4_-10μg/ml (Fig 3A, Table 1; cutoff at 2 fold changes (log_2_Ratio≥1 or ≤-1); red color indicating up regulation, black color means down regulation). A total of 252 genes were overlapping in these two treatments, 54 genes were specifically regulated by ZnO-NP-5μg/ml and 1116 genes were changed just by ZnSO_4_-10μg/ml.

**Table 1:** Summary of RNA-seq and iTRAQ data.

Groups	Type	Number of Proteins	Number of Genes	Number of correlations
Ctrl-VS-ZnO-NP-5	Identification	4418	13537	4272
Ctrl-VS-ZnO-NP-5	Quantitation	3170	13537	3062
Ctrl-VS-ZnO-NP-5	Differential Expression	191	306	16
Ctrl-VS-ZnSO4-10	Identification	4418	13829	4274
Ctrl-VS-ZnSO4-10	Quantitation	3170	13829	3065
Ctrl-VS-ZnSO4-10	Differential Expression	290	1368	65

The differentially expressed genes were classified into a few groups in each of three ontologies biological process, cellular component and molecular function by Gene Ontology Functional Classification (S1 Fig). Although ZnO-NP-5μg/ml altered less number of genes, the percentage of genes (compared to total genes altered in each treatment) changed for each terms by ZnO-NP-5μg/ml and ZnSO4-10μg/ml treatments was similar. There was just slight difference between these two treatments for some of the terms: immune system process (in biological process ontology), antioxidant activity (in molecular function ontology) (S1 Fig). Since we were more interested in the reproductive toxicity of ZnO NPs, the genes related to development, reproduction and growth were grouped together for each treatment. Totally 316 differentially regulated genes were involved in development, reproduction and growth, and twelve of them (3 up-regulated and 9 down-regulated) were just altered by ZnO-NP-5μg/ml, 75 of them (65 up-regulated and 10 down-regulated) were changed by both ZnO-NP-5μg/ml and ZnSO_4_-10μg/ml, and 229 of them (205 up-regulated and 24 down-regulated) were regulated solely by ZnSO_4_-10μg/ml. And most (75%) of the genes altered solely by ZnO-NP-5μg/ml and about 12% of genes changed just by ZnSO_4_-10μg/ml were down regulated (Fig 3B). This suggested that NPs produced more profoundly negative effects on gene expression than Zn^2+^.

**Fig 4:**
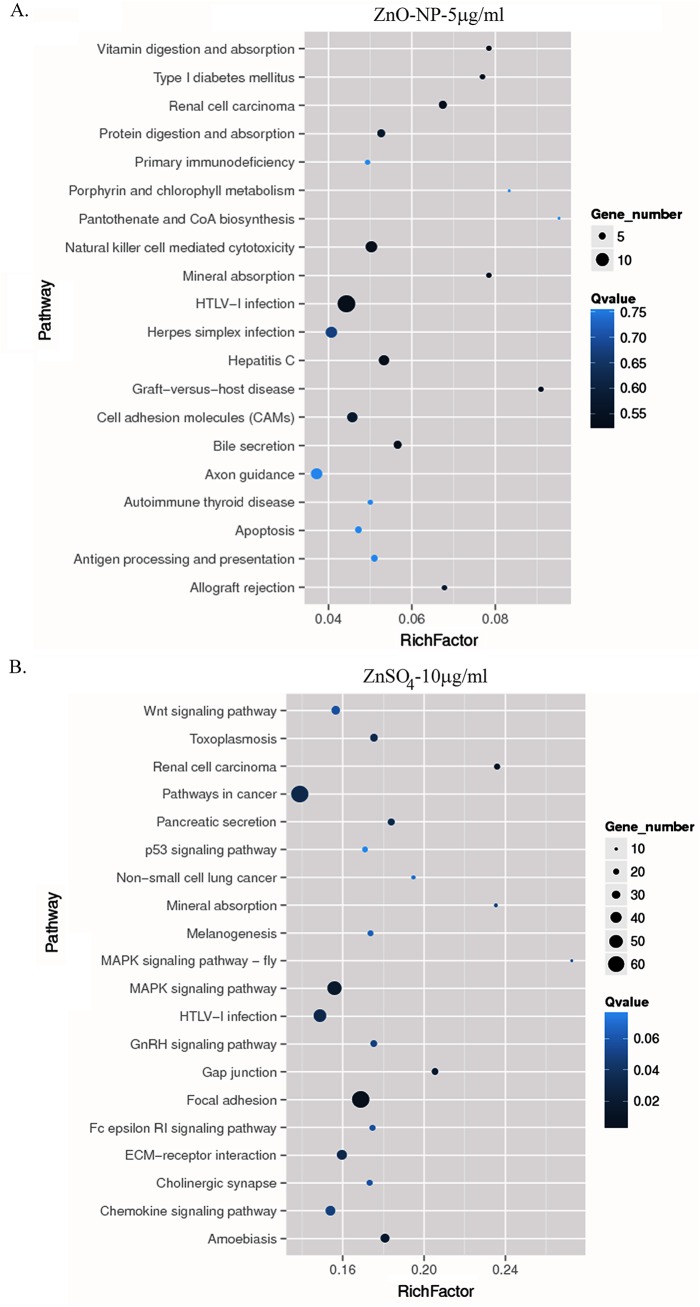
The top 20 pathways in KEGG enrichment analyses. A. The top 20 pathways in KEGG analysis for ZnO-NP-5μg/mL treatment; and B. The top 20 pathways in KEGG analysis for ZnSO4-10μg/mL treatment. Different size of the dots meant the different number of genes in each pathway (more number of genes with larger dot), and the color means the Q-Value.

Based on KEGG pathway enrichment analysis, the top 20 pathways in each treatment were shown in Fig 4. The greatest numbers of genes were classified in HTLV-I infection pathway, and then the greater numbers of genes were classified into natural killer cell mediated cytotoxicity, cell adhesion molecules in ZnO-NP-5μg/ml treatment. The greatest numbers of genes were classified in focal adhesion pathway, pathways in cancer, and then the greater numbers of genes were classified into MAPK signaling pathway in ZnSO_4_-10μg/ml treatment. Because less numbers of genes were altered by ZnO-NP-5μg/ml, the Qvalue was much higher while the Rich Factor was much lower in this treatment than that in ZnSO4-10μg/ml treatment.

RNA seq results were confirmed by q-RT-PCR analysis. Total 26 genes were randomly selected from the differentially expressed genes for quantification. RNA-seq and q-RT-PCR data matched very well for all tested genes (Table 2).

**Table 2:** mRNA q-RT-PCR data.

		ZnO-NP-5		ZnSO_4_-10	
		Fold Change	p-value	Fold Change	p-value
mRNA	Symbol				
	S1PR1	**—**		**2.88**	0.002
	CRIP1	**—**		**2.27**	0.011
	TGFBR2	**-2.88**	0.046	**—**	
	CA10	**-2.8**	0.003	**—**	
	CHRDL1	**-2.46**	0.006	**—**	
	MPZL2	**-2.34**	0.004	**—**	
	SEMASA	**-2.5**	0.022	**—**	
	CHRNA7	**-2.04**	0.005	**—**	
	DPYSL2	**2.09**	0.048	**3.28**	0.006
	OSGIN1	**2.06**	0.037	**4.52**	0.001
	CCK	**2.09**	0.039	**19.47**	0.018
	PDGFB	**2.11**	0.039	**2.12**	0.007
	LGALS1	**—**		**2.36**	0.032
	GDA	**3.43**	0.006	**16.95**	0.001
	CSRP2	**—**		**—**	
	PIWIL1	**—**		**—**	
	DDX4	**—**		**—**	
	KCTD15	**12.38**	0.001	**20.68**	0.001
	MAP2K3	**—**		**2.53**	0.020
	TEX14	**1.8**	0.048	**7.68**	0.001
	SLC30A1	**2.04**	0.016	**3.54**	0.001
	MGAT3	**-1.7**	0.017	**—**	
	TXN	**2**	0.002	**2.86**	0.001
	GCLC	**1.92**	0.041	**3.17**	0.016
	STK31	**—**		**—**	
	VTG2	**—**		**—**	

### Effects of ZnO NPs on Protein Expression

**Fig 5:**
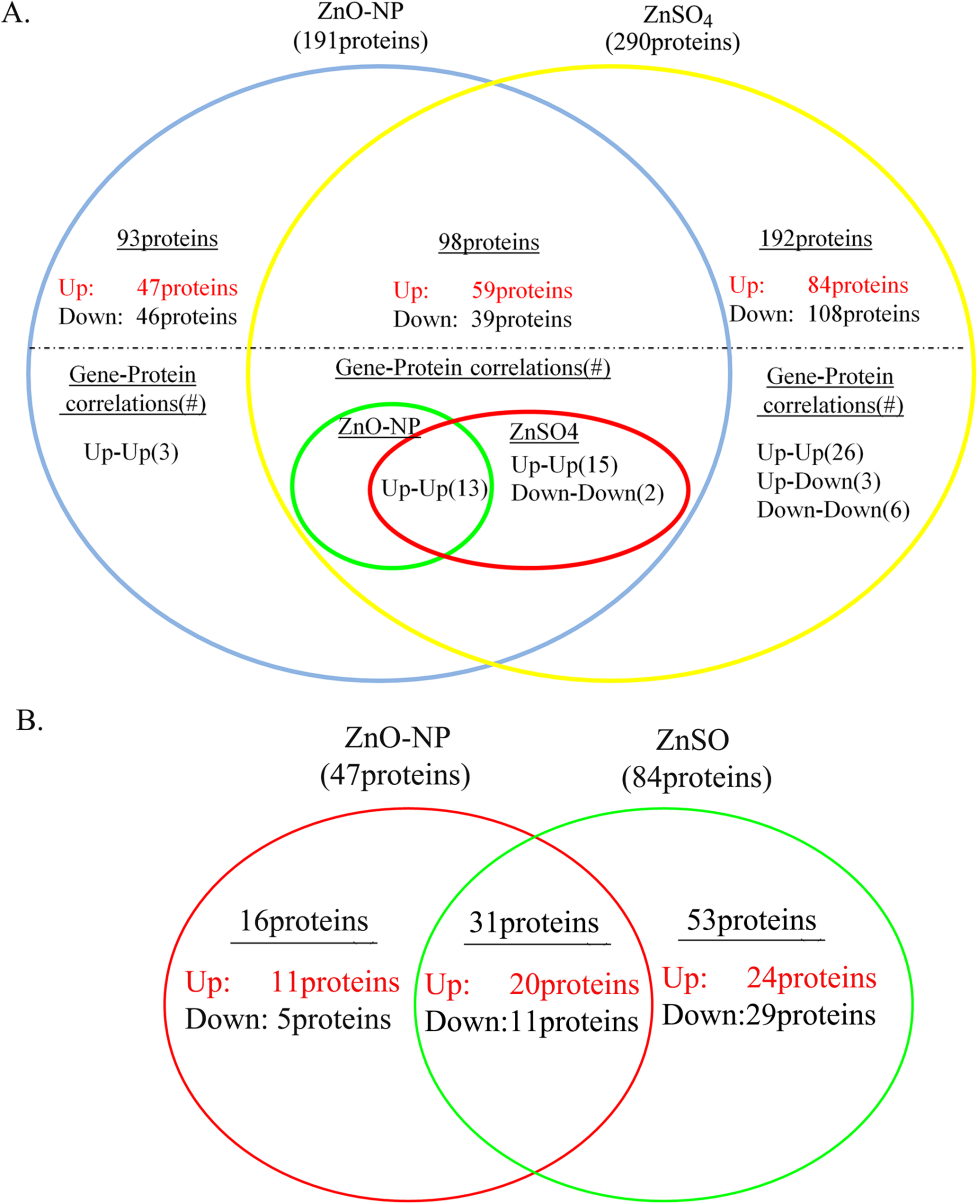
Effects of ZnO-NP-5μg/mL and ZnSO4-10μg/mL treatments on protein expression in GCs. A. Summary of number of proteins regulated by ZnO-NP-5μg/mL and/or ZnSO4-10μg/mL, and the same sets of proteins regulated by the two treatments, and the different sets of proteins regulated by each treatment. B. The regulated proteins related to development, growth and reproduction by these two treatment groups. The red color meant the proteins up-regulated, black meant the proteins down-regulated. The number was fold change. They were significant regulated.

4418 proteins were identified and 3170 proteins were quantified in this investigation (Table 1). A total of 98 proteins were regulated by both ZnO-NP-5μg/ml and ZnSO_4_-10μg/ml treatments, 93 proteins were specifically regulated just by ZnO-NP-5μg/ml, and 192 proteins were changed solely by ZnSO_4_-10μg/ml (Fig 5A, Table 1; red color indicated up regulation, black color meant down regulation).

The differentially expressed proteins were enriched into a few groups in each of three ontologies biological process, cellular component and molecular function by Gene Ontology (GO) Enrichment analysis (S2, S3, S4, S5, S6 and S7 Figs). In cellular component ontology the differentially expressed proteins were enriched into groups: cell, macromolecular complex, extracellular region, extracellular matrix, cell junction in ZnO-NP-5μg/ml treatment (S2 Fig) while the proteins were enriched into groups: membrane-enclosed lumen, organelle, cell, macromolecular complex in ZnSO_4_-10μg/ml treatment (S3 Fig). In molecular function ontology the differentially expressed proteins were enriched into groups: binding, oxidoreductase activity, catalytic activity, antioxidant activity and transporter activity in ZnO-NP-5μg/ml treatment (S4 Fig) while the proteins were enriched into groups: binding, catalytic activity and transporter activity in ZnSO_4_-10μg/ml treatment (S5 Fig). In biological process ontology the differentially expressed proteins were enriched into similar groups in both ZnO-NP-5μg/ml and ZnSO_4_-10μg/ml treatments: metabolic process, biological regulation, cellular process, cellular component organization or biogenesis, developmental process, single organism process, signaling, localization, reproduction, multicellular organismal process, response to stimulus, biological adhesion, multi-organism process, establishment of localization and immune system process (S6 and S7 Fig). Because we were more interested in the reproductive toxicity of ZnO NPs, the proteins involved in development, reproduction and growth were grouped together for comparison. Of the proteins involved in development, reproduction and growth, sixteen proteins were changed solely by ZnO-NP-5μg/ml (11 up regulated and 5 down regulated), 31 proteins were altered by both ZnO-NP-5μg/ml and ZnSO_4_-10μg/ml (20 up regulated and 11 down regulated), and 53 proteins were regulated by ZnSO_4_-10μg/ml only (24 up regulated and 29 down regulated) (Fig 5B).

Based on KEGG pathway enrichment analysis, the top pathways with more than 5 proteins in each pathway in ZnO-NP-5μg/ml or ZnSO_4_-10μg/ml treatment were listed in Table 3. There are some pathways in common for ZnO-NP-5μg/ml and ZnSO_4_-10μg/ml treatments (Highlight in pink), and also there are specific pathways for each treatment alone. The greatest numbers of proteins were classified into metabolic pathways for both treatments.

**Table 3:** KEGG enrichment for differentially expressed proteins.

Treatments	Terms	# of protein	Terms	# of protein	Terms	# of protein
ZnO-NP	Metabolic pathways	30	Focal adhesion	6	Dilated cardiomyopathy	5
	Regulation of actin cytoskeleton	7	Endocytosis	6	Cell cycle	5
	Pathogenic Escherichia coli infection	6	Fructose and mannose metabolism	5	Adherens junction	5
	Phagosome	6	Amoebiasis	5	Leukocyte transendothelial migration	5
	Epstein-Barr virus infection	6	Hypertrophic cardiomyopathy (HCM)	5	Lysosome	5
						
ZnSO4	Metabolic pathways	57	Bacterial invasion of epithelial cells	7	Fc epsilon RI signaling pathway	5
	Focal adhesion	16	Oxidative phosphorylation	7	Arginine and proline metabolism	5
	Pathways in cancer	14	Parkinson's disease	7	Pyruvate metabolism	5
	Regulation of actin cytoskeleton	13	RNA transport	7	VEGF signaling pathway	5
	Leukocyte transendothelial migration	10	Glutathione metabolism	6	Arrhythmogenic right ventricular cardiomyopathy (ARVC)	5
	Tight junction	10	Adipocytokine signaling pathway	6	PPAR signaling pathway	5
	Amoebiasis	10	Osteoclast differentiation	6	Shigellosis	5
	Purine metabolism	9	Small cell lung cancer	6	Fc gamma R-mediated phagocytosis	5
	MAPK signaling pathway	9	Adherens junction	6	Insulin signaling pathway	5
	Huntington's disease	9	Chemokine signaling pathway	6	Ribosome	5
	Alzheimer's disease	8	Phagosome	6	HTLV-I infection	5
	Pyrimidine metabolism	7	Cysteine and methionine metabolism	5	Epstein-Barr virus infection	5
	Toxoplasmosis	7	ECM-receptor interaction	5	Protein processing in endoplasmic reticulum	5
	Pathogenic Escherichia coli infection	7	Citrate cycle (TCA cycle)	5	Endocytosis	5

**Fig 6:**
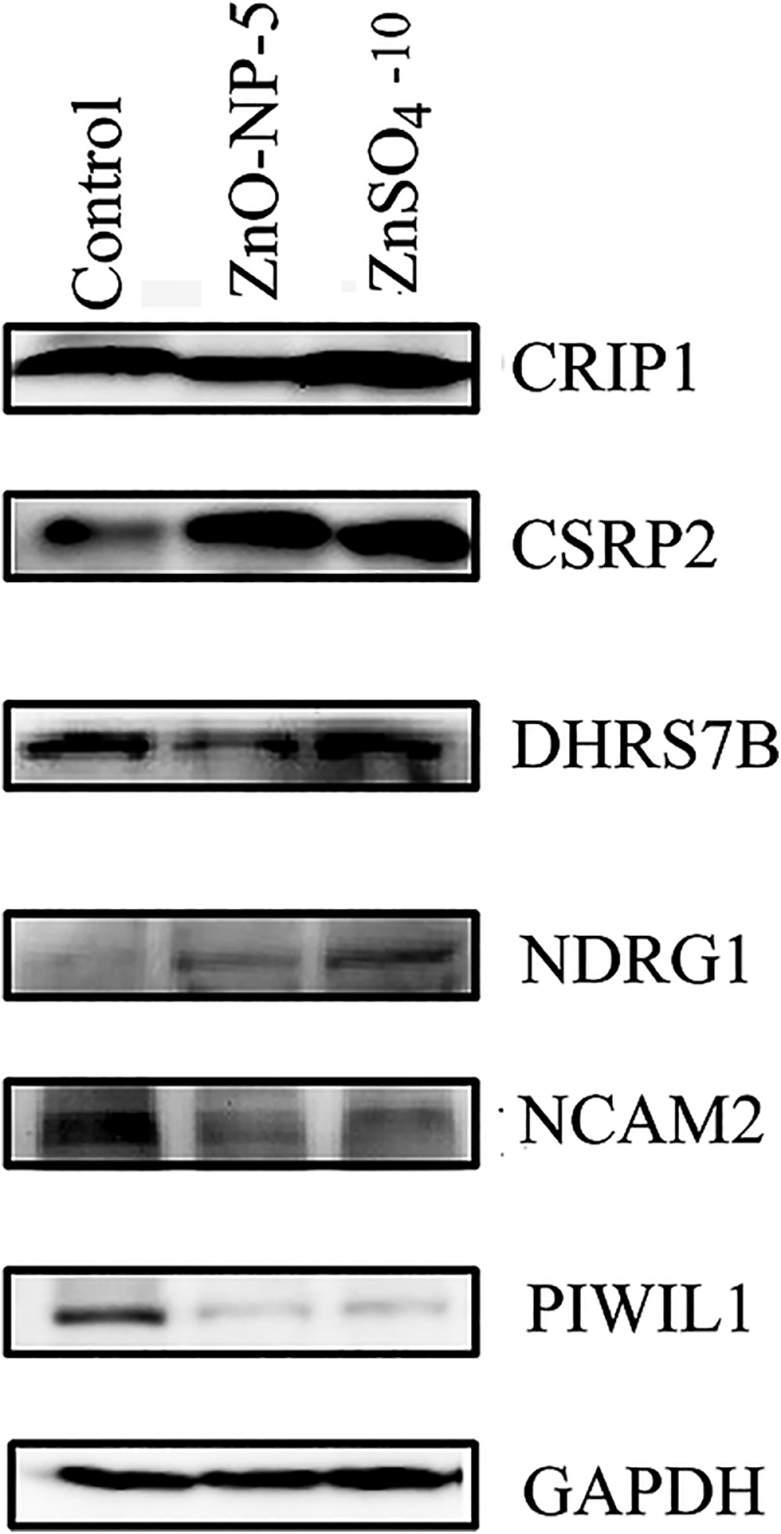
Western blotting images of 6 protein regulated by ZnO-NP-5μg/mL and/or ZnSO4-10μg/mL.

Western blotting was used to confirm the iTRAQ data. Eight proteins were examined and six primary antibodies worked in GCs (Fig 6). The western blotting results matched well with iTRAQ data except that NCAM2 in ZnSO_4_-10μg/mL treatment was decreased by western blotting detection, while it was not changed by iTRAQ analysis.

### Gene-Protein Correlation

Gene and protein correlation was low (Fig 5A, Table 1). Of the 306 genes and 191 proteins regulated by ZnO-NP-5μg/mL, sixteen gene-protein pairs were correlated. Of the 1368 genes and 290 proteins regulated by ZnSO_4_-10μg/mL, 65 gene-protein pairs were correlated. There were just two gene-protein pairs involved in development, reproduction and growth in ZnO-NP-5μg/ml treatment. There were 12 gene-protein pairs related to development, reproduction and growth in ZnSO_4_-10μg/ml treatment.

### Cell Cycle Distributions Unchanged by ZnO-NPs-5μg/ml While Altered by ZnSO_4_-10μg/ml

**Fig 7:**
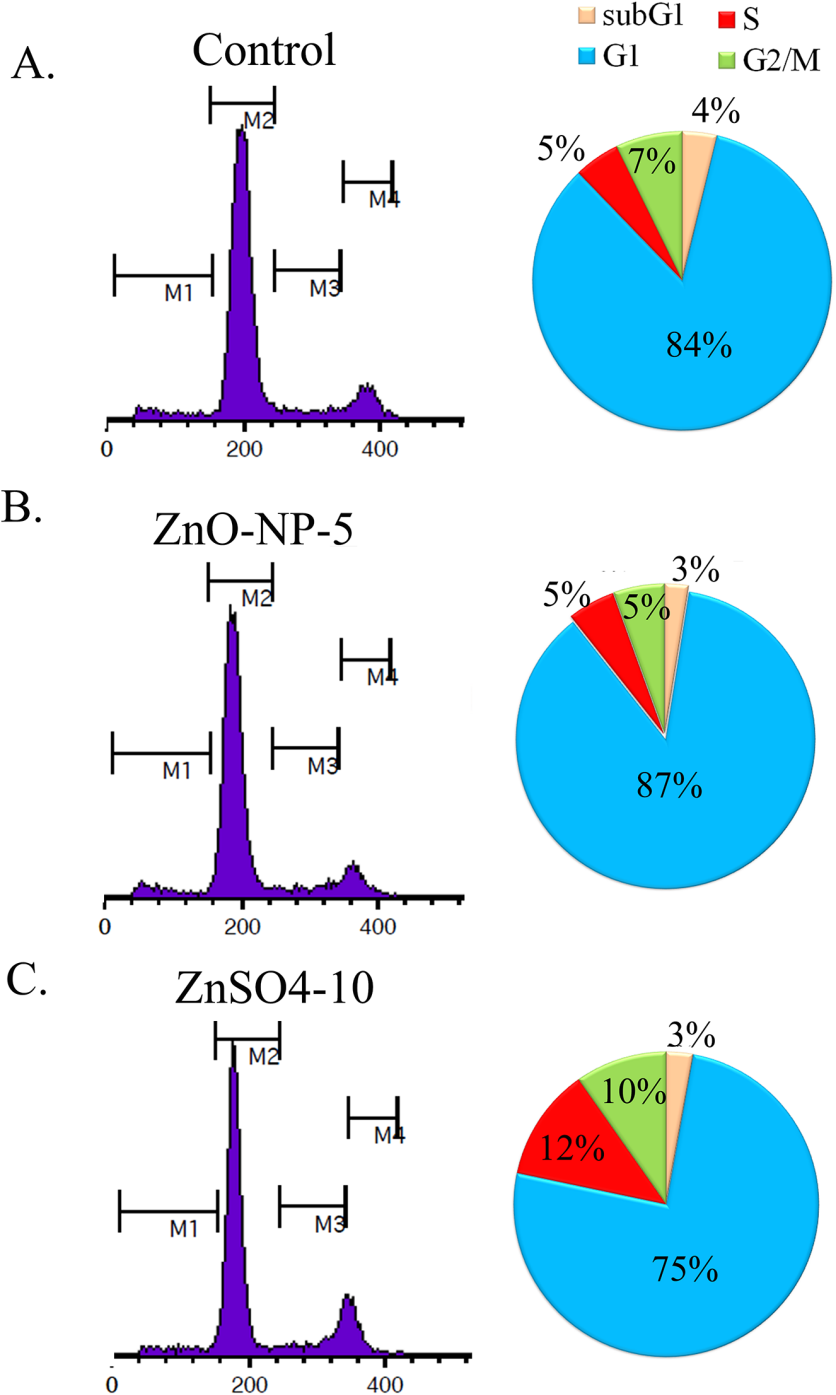
Cell cycle distributions in ZnO-NP-5μg/mL or ZnSO4-10μg/mL treatment. A. Cell cycle distributions in control GCs; B. Cell cycle distributions in ZnO-NP-5μg/mL treated GCs; C. Cell cycle distributions in ZnSO4-10μg/mL treated GCs.

Although ZnO-NP-5μg/ml and ZnSO_4_-10μg/ml produced same amount of Zn in the treated cells and caused similar cell growth inhibition, ZnSO_4_-10μg/ml altered the expression of more number of genes and proteins. This indicated that ZnSO_4_-10μg/ml should change the genetic material differentially compared to ZnO-NP-5μg/ml. The cell cycle distributions in these treatments were shown in Fig 7. Compared to control, ZnO- NP-5μg/ml did not change the cell cycle distributions while ZnSO_4_-10μg/ml increased the number of cells in S and G2/M phases. This indicated that ZnSO_4_-10μg/ml promoted the cell proliferation while ZnO-NP-5μg/ml did not. This helped to explain why ZnSO_4_-10μg/ml changed the expression of more number of genes and proteins compared to ZnO-NP-5μg/ml.

## Discussion

Numerous studies have reported that ZnO NP posed adverse effects on organisms and even on reproduction [8–11]. It was found that ZnO NPs reduced number of born/live pups, decreased the body weight of the pups and increased the fetal resorption when the rats were treated with ZnO NPs, however, no mechanistic insight of the effects [11]. Talebi et al. reported that ZnO NP caused testicular toxicity including changes in the sperm number, motility and percentage of abnormality in male mice however the mechanistic mechanisms were not provided either [12]. The goal of this investigation was to explore the molecular mechanisms of ZnO NPs on female reproductive systems. Our results indicated that NPs and Zn^2+^ are different. Starting at 5μg/ml of ZnSO_4_ gradually inhibited the growth of GCs. However, ZnO-NP-1μg/ml began to inhibit the growth of GCs and the inhibition increased very quickly. It was very interesting to note that ZnO-NP-5μg/ml and ZnSO_4_-10μg/ml caused similar cell growth inhibition with same intracellular Zn concentrations. The previous report has already found that NPs were easily absorbed into biological systems because of the small size [31]. Although early studies have already used TEM to find the nanoparticles in the biological samples [23], the NPs used in those studies were not confirmed. In this investigation, TEM was applied to find the particles in the cells and at the same time EDS was used to confirm the particles made up of Zn. It is very common to observe numerous NPs-like dark particles in the biological samples since the biological samples are very complex. Therefore it is very difficult to really discern which particles are the NPs in the biological samples if EDS is not used to confirm the metal element of the NPs used.

Quantitative proteomics have not been applied yet while quantitative genes expression has been used in ZnO NPs research. Tuomela et al. have found that ZnO NPs modulated the expression of genes related to cell death and growth in human cell lines [14]. Poynton et al. have reported that ZnO NPs affected the expression of genes involved in cytoskeletal transport, cellular respiration, and reproduction in *Daphnia magna* [9]. In this investigation, although ZnO-NP-5μg/mL and ZnSO_4_-10μg/mL treatments produced similar intracellular Zn contents, the number and profiling of regulated genes were different in these two treatments. NPs played important roles for these differences, and different amounts of Zn^2+^ in ZnO-NPs-5 μg/mL or ZnSO_4_-10 μg/mL treatment might also make some contribution. Quantitative protein analysis (iTRAQ) revealed that 191 and 290 proteins were regulated by ZnO-NP-5μg/ml and ZnSO_4_-10μg/ml, respectively. 93 proteins (48.69% (93/191)) were changed just by ZnO-NP-5μg/ml and 192 proteins (66.20% (193/290)) were regulated by ZnSO_4_-10μg/ml only. However, of the 306 genes modulated by ZnO-NP-5μg/ml, only 17.64% (54 out of 306) genes were regulated solely by ZnO-NP-5μg/ml; of the 1368 genes altered by ZnSO_4_-10μg/ml, 81.58% (1112 out of 1368) genes were regulated just by ZnSO4-10μg/ml. This indicated that ZnO-NP-5μg/ml had more impact on protein expression than gene expression however ZnSO4 regulated more gene expression than protein expression although they produced same amount of Zn in the cells. These results matched well with cell cycle data that ZnSO4-10μg/ml resulted in more cells in S and G2/M phases (genetic materials increased). Gene Ontology (GO) is a useful tool to describe the function of the genes or proteins. The proteins regulated by ZnO-NP-5μg/ml were specifically grouped to extracellular region, extracellular matrix and cell junction by cell component ontology classification which suggested that NPs affected the cell surface and cell-cell interaction. The differentially expressed proteins were specifically enriched into oxidoreductase activity and antioxidant activity in ZnO-NP-5μg/ml treated cells by molecular function ontology classification which indicated that NPs might increase cell oxidative stress. Early studies have found that ZnO NPs produced ROS in the treated cells [26]. ROS was analyzed in the cells, however, there was no difference with ROS between ZnO-NP-5μg/ml and control or ZnSO_4_-10μg/ml treated cells (Data not shown). This may be because the concentration of ZnO NPs used in this study was low which might just increase a little amount of ROS.

In this study, we are more interested in the biological process category (GO) for development, growth and reproduction, because one of our focuses is the impact of ZnO NPs on reproductive system. Of the 54 genes specifically regulated by ZnO-NP-5μg/mL, 12 (22.2%) were related to development, and of the 1116 genes specifically regulated by ZnSO_4_-10μg/mL, 229 genes (20.5%) were related to development. Totally 100 proteins related to development, growth or reproduction were altered by ZnO-NP-5μg/mL and/or ZnSO_4_-10μg/mL treatments. 17.2% (16 out of 93) and 27.6% (53 out of 192) of the proteins involved in development, growth or reproduction were regulated specifically by ZnO-NP-5μg/mL or ZnSO_4_-10μg/mL, respectively. Of these proteins, CRIP1, CRSP2, PIWIL1, DDX4, NGRD1 and VTG2 have very important functions in development and reproduction. CRIP (cycteine-rich intestinal protein), a zinc-binding protein, has initially been determined as a developmentally regulated intestinal gene and has subsequently been found in several other tissues and cells [32]. It is involved in cellular growth and differentiation and has been reported to be a novel biomarker for a few cancers. The CRIP protein was down-regulated by ZnO-NP-5μg/mL however up-regulated by ZnSO_4_-10μg/mL which suggested that ZnO NPs specifically regulated this protein expression. DDX4, the DEAD box protein family DDX4 (VASA), is an ATP-dependent RNA helicase expressed in the germ cells of all animals germ cell. It is involved in cell development and is the most widely used marker of the germ cell lineage [33]. It was down-regulated by ZnSO_4_-10μg/mL however not changed by ZnO-NP-5μg/mL which indicated that Zn^2+^ rather than NPs regulated this protein expression. PIWI proteins, essential for mouse spermatogenesis and needed for the biogenesis of piRNAs, are involved in germ line development of many metazoan species [34]. In this study, PIWIL1 protein was down-regulated in both treatment groups. The cysteine and glycine rich protein 2 (CSRP2), a LIM domain protein, is expressed in the vascular system and is supposed to have a critically role for the development or maintenance of vital processes within organisms [35]. CSRP2 was up-regulated by both ZnO-NP-5μg/mL and ZnSO_4_-10μg/mL treatments. N-myc downstream regulated 1 gene (*NDRG1)*, also known as *Drg1*, *Cap43*, *Rit42*, *RTP* and *PROXY-1*, is involving in cell growth and differentiation [36]. It was up-regulated by these two treatment groups. Vitellogenin (VTG) is a precursor of the yolk protein vitellin. It is a lipoglycoprotein that is employed as a vehicle to provide the developing embryo with proteins, lipids, carbohydrates, and other essential resources. VTG2 was increased by ZnO-NP-5μg/mL however not changed in ZnSO_4_-10μg/mL group which further suggested NPs specifically regulated protein expression [37].

## Conclusion

Intact NPs were found in the treated GCs and ZnO-NP-5μg/ml treatment produced the same amount of intracellular Zn and resulted in similar cell growth inhibition compared with ZnSO_4_-10μg/ml treatment. However, ZnO-NP-5μg/ml specifically regulated the expression of genes and proteins compared to that in ZnSO_4_-10μg/ml treatment. For the first time, this investigation reported that intact NPs produced different impact on the expression of proteins involved in specific pathways compared to that by Zn^2+^. Because of the small size and broad applications, ZnO NPs might enter into our body and pose adverse effects on the female reproductive systems through regulation of specific signaling pathways. Actually, in our anther study, NPs were detected in hens’ ovaries when the hens were exposed to low concentration of ZnO nanoparticles in their diet. This raises the health concern that ZnO NPs may produce adverse effect on female reproductive systems. Further studies on how ZnO NPs specifically regulate genes and proteins need to be explored.

## Supporting Information

S1 Fig GO classifications for genes regulated by ZnO-NP-5μg/ml or ZnSO4-10μg/ml treatment. A. GO classifications for the genes regulated by ZnO-NP-5μg/ml treatment; B. GO classifications for the genes regulated by ZnSO4-10μg/ml treatment. BP: biological process; CC: cellular component; MF: molecular function.
(TIF)

S2 Fig GO classification of cellular component for the proteins regulated by ZnO-NP-5μg/ml treatment. (TIF)

S3 Fig GO classification of cellular component for the proteins regulated by ZnSO4-10μg/ml treatment. (TIF)

S4 Fig GO classification of molecular function for the proteins regulated by ZnO-NP-5μg/ml treatment. (TIF)

S5 Fig GO classification of molecular function for the proteins regulated by ZnSO4-10μg/ml treatment. (TIF)

S6 Fig GO classification of biological process for the proteins regulated by ZnO-NP-5μg/ml treatment. (TIF)

S7 Fig GO classification of biological process for the proteins regulated by ZnSO4-10μg/ml treatment. (TIF)

S1 Table Primers for Q-RT-PCR. (DOC)

S2 Table Raw data of RNA-seq analysis. (XLS)

S3 Table Raw data of iTRAQ analysis. (XLS)
