# Supplementary figures and images for: Differential Regulation of Gene and Protein Expression by Zinc Oxide Nanoparticles in Hen’s Ovarian Granulosa Cells: Specific Roles of Nanoparticles
Source: PLoS One. 2015 Oct 13;10(10):e0140499. doi: 10.1371/journal.pone.0140499 (PMC4604165; doi:10.1371/journal.pone.0140499)

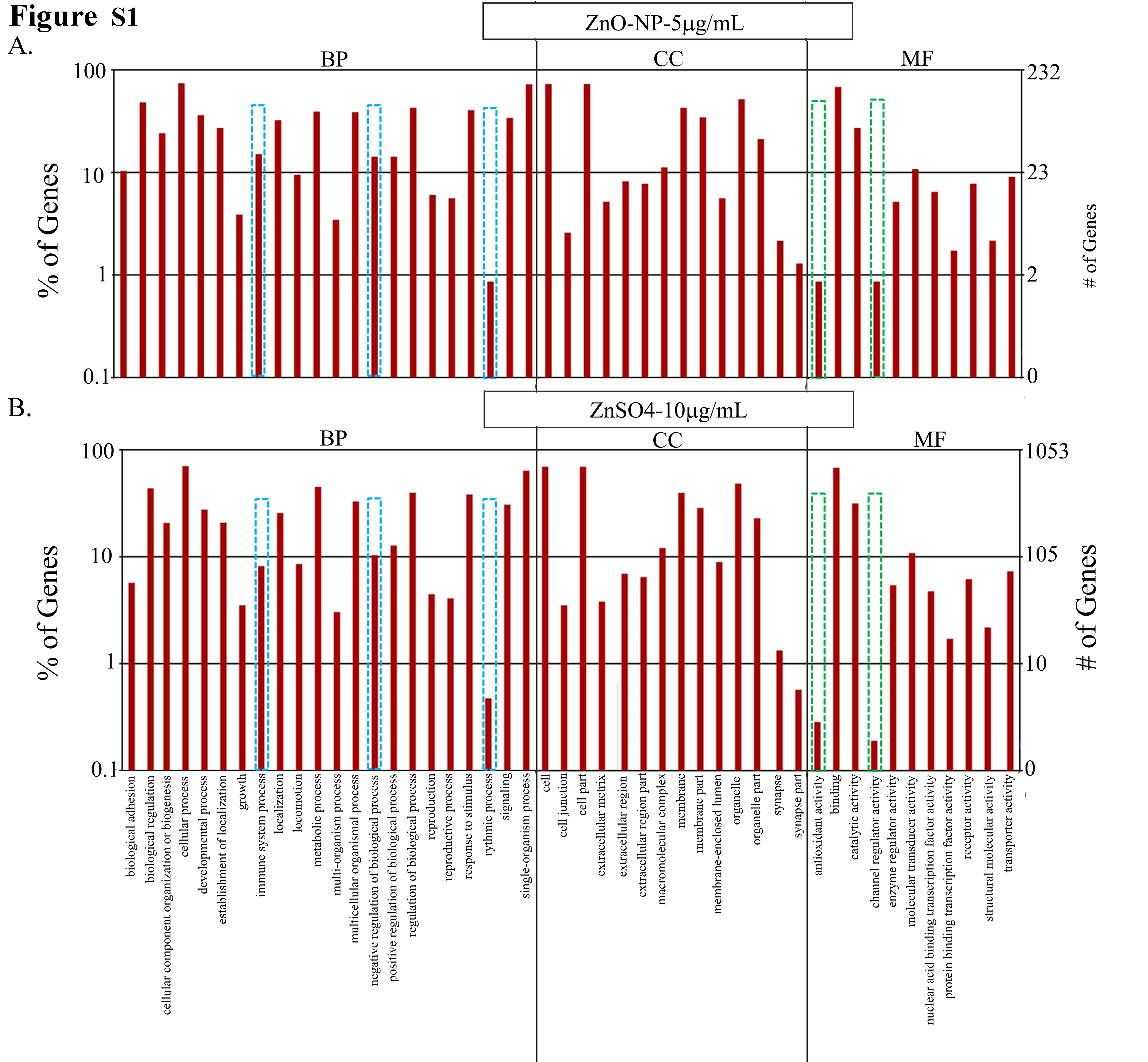

Supplement: S1 Fig — A. GO classifications for the genes regulated by ZnO-NP-5μg/ml treatment; B. GO classifications for the genes regulated by ZnSO4-10μg/ml treatment. BP: biological process; CC: cellular component; MF: molecular function. (TIF) [file pone.0140499.s001.tif]
