# Supplementary material for: Differential Regulation of Gene and Protein Expression by Zinc Oxide Nanoparticles in Hen’s Ovarian Granulosa Cells: Specific Roles of Nanoparticles
Source: PLoS One. 2015 Oct 13;10(10):e0140499. doi: 10.1371/journal.pone.0140499 (PMC4604165; doi:10.1371/journal.pone.0140499)
Supplement: S1 Table — (DOC) [file pone.0140499.s008.doc]

**S1Table Primer sequencing for q-RT-PCR.**

| Gene | Site | Sequence |
| --- | --- | --- |
| S1PR1 | Forward | GCTGAGGAGCGAGTATCATTAG (Sense) |
|  | Reverse | ACATGCTCCAGGGAAATAGAC (Anti-sense) |
| CRIP1 | Forward | AGCCACACGTTCAAGTAGAG (Sense) |
|  | Reverse | CAGAGTGATGAAGGACGAGAAG (Anti-sense) |
| TGFBR2 | Forward | CCTTCAACATGGAGCTCTACAA (Sense) |
|  | Reverse | GCTTTAGTCACAGACACCTCTAC (Anti-sense) |
| CA10 | Forward | GGAGGTTCAACTGATCCACTAC (Sense) |
|  | Reverse | GGAAACTACCACCAACCCATTA (Anti-sense) |
| CHRDL1 | Forward | TCCCAGGATAACTCTCCATCTC (Anti-sense) |
|  | Reverse | CAAACCAGGGCCAGACTAAA (Sense) |
| MPZL2 | Forward | CCGAGATGAGTGACTGGATAGA (Sense) |
|  | Reverse | CAATAATCTGGCAGCACAAAGG (Anti-sense) |
| SEMASA | Forward | AACTGGAAAGCTTCAGGATAGG (Sense) |
|  | Reverse | CCTCCACCACCCTCATAATTC (Anti-sense) |
| CHRNA7 | Forward | AGTAACCATGAGACGCAGAAC (Sense) |
|  | Reverse | TGCTGGAAGCAGAAAGACTAAT (Anti-sense) |
| DPYSL2 | Forward | GACTGGCAAGATGGATGAGAA (Sense) |
|  | Reverse | ATCAGAGCCCACTGCAATAC (Anti-sense) |
| OSGIN1 | Forward | CTCTCTTCATCCTCATCCCATTC (Sense) |
|  | Reverse | GCTGTTGGATCTTCCCTCTAAA (Anti-sense) |
| CCK | Forward | CTACATGGGCTGGATGGATTT (Sense) |
|  | Reverse | GTACAGACATGGGAGTGTCATT (Anti-sense) |
| PDGFB | Forward | AAACCATGTGTCCCTGTCTC (Sense) |
|  | Reverse | CATGTCACGGGAGATCTCAAA (Anti-sense) |
| LGALS1 | Forward | ATCAACCCAAGCGATCTGAC (Sense) |
|  | Reverse | GTGAAGTCTCCATGCGTATCA (Anti-sense) |
| GDA | Forward | CAACAGAGGCGAGATACAAAGA (Sense) |
|  | Reverse | TGCGAAGTAGCAAGCTGTAG (Anti-sense) |
| CSRP2 | Forward | TGCTTCCTCTGCATGGTTT (Sense) |
|  | Reverse | GGGCCGTACTTCTTTCCATAG (Anti-sense) |
| PIWIL1 | Forward | CTCCACCACTGACAGAAGAAC (Sense) |
|  | Reverse | GAAACCCTGCTGAAATCTGTAATC (Anti-sense) |
| DDX4 | Forward | CAAGCAGGGCGGGATTTA (Sense) |
|  | Reverse | GCTTGCAGTTACACCATCTTTC (Anti-sense) |
| KCTD15 | Forward | GCCACCTCTTGTTGTTCATTTA (Sense) |
|  | Reverse | CATCAGACAGTGGAAGGTAGAG (Anti-sense) |
| MAP2K3 | Forward | GAACACTCAGGAGCAGAAGAG (Sense) |
|  | Reverse | CGGAATAGGGCTCCATAGAAAG (Anti-sense) |
| TEX14 | Forward | GGGAGTGACTCACATTCTTCAG (Sense) |
|  | Reverse | CATCCTTCTCTCCTCGCTTATTT (Anti-sense) |
| SLC30A1 | Forward | CTGCCAACCTACACCTTCATAG (Sense) |
|  | Reverse | CAGGGTCAGGGTTTCAGATTAG (Anti-sense) |
| MGAT3 | Forward | GGATGAAGATGAGACGCCATAA (Sense) |
|  | Reverse | GGGAAGGTGACATAGGAAAGG (Anti-sense) |
| TXN | Forward | CCAGTTCTACAAGAACGGAAAGA (Sense) |
|  | Reverse | TCCATGGCGGGAGATTAGA (Anti-sense) |
| GCLC | Forward | CAGTTCTGGCAGCATGTTTATTC (Sense) |
|  | Reverse | CTCCATGATCTCCCTCCATCTA (Anti-sense) |
| STK31 | Forward | CCCAGTGCTTAACAGAGGTTT (Sense) |
|  | Reverse | CTCCTTTCTGTGAGCTCCTTTC (Anti-sense) |
| VTG2 | Forward | GGAGAACCAGCCAGCTTAAA (Sense) |
|  | Reverse | AAGGCCGTTATGGCATCTATC (Anti-sense) |
| GAPDH | Forward | GGCATTGTGGAGGGTCTTAT (Sense) |
|  | Reverse | ACGCTGGGATGATGTTCTG (Anti-sense) |
| Actin-b | Forward | CCCTGGAGAAGAGCTACGA (Sense) |
|  | Reverse | GGAAAGAGGGTTGGAACAGAG (Anti-sense) |
